# Clinical evaluation of a novel plasma pTau217 electrochemiluminescence immunoassay in Alzheimer’s disease

**DOI:** 10.1038/s41598-024-51334-x

**Published:** 2024-01-05

**Authors:** Pia Kivisäkk, Hadia A. Fatima, Danielle S. Cahoon, Brunah Otieno, Leena Chacko, Farnaz Minooei, Catherine Demos, Martin Stengelin, George Sigal, Jacob Wohlstadter, Steven E. Arnold

**Affiliations:** 1 Alzheimer’s Clinical and Translational Research Unit, Department of Neurology, Massachusetts General Hospital, Harvard Medical School, 114 16th Street, Room 2300, Charlestown, Boston, MA 02129 USA; 2 Meso Scale Diagnostics, LLC., Rockville, MD USA

**Keywords:** Alzheimer's disease, Diagnostic markers

## Abstract

A growing literature suggests that plasma levels of tau phosphorylated at amino acid 217 (pTau217) performs similarly to cerebrospinal fluid (CSF) biomarkers and PET imaging to detect amyloid pathology and to provide diagnostic and prognostic information in Alzheimer’s disease (AD), but a significant limiting factor thus far has been a lack of widely available immunoassays. We evaluated a novel pTau217 S-PLEX® assay developed by Meso Scale Discovery (MSD; Rockville, MD) in plasma from 131 individuals with AD confirmed by CSF biomarkers and controls. Technical performance of the assay was excellent with an LLOQ of 1.84 pg/mL and intra/interplate CVs of 5.5% (0.3–15.0%) and 5.7% (range 0.3–13.4%), respectively. The pTau217 plasma assay differentiated AD and controls with an AUC of 0.98 (95% CI 0.96–1.0) and pTau217 levels were 3.9-fold higher in individuals with AD. This performance was significantly better than what was observed for plasma pTau181, performed in parallel, and comparable to published data on existing pTau217 assays. While further clinical validation and head-to-head comparisons are needed to fully establish the role for the novel pTau217 S-PLEX assay, these data demonstrate the utility of the assay to detect AD pathology.

## Introduction

The rapid development of immunoassays sensitive enough to detect Alzheimer’s disease (AD) biomarkers with high accuracy in plasma has dramatically changed the utility of blood-based biomarkers in AD, allowing for early and minimally invasive detection of disease pathology. While amyloid-ß42 (Aß42), either as a ratio with Aß40 (Aß42/40) or tau phosphorylated at amino acid 181 (pTau181/Aß42), remains the most commonly used biomarker in cerebrospinal fluid (CSF) to identify AD pathology^1,2^, its use as a blood-based biomarker has been more limited. It is generally believed that peripheral contributions of Aß dilute the AD-specific changes, resulting in small fold-differences in Aß42 levels between AD and controls in plasma and rendering Aß42 a less robust biomarker for AD pathology^3^. The performance of Aß42 immunoassays in plasma is furthermore lagging behind more sensitive immunoprecipitation mass spectrometry (IP-MS) methods^4^, which remain expensive and with limited accessibility. Tau phosphorylated at different epitopes (pTau) have instead emerged as the most promising blood-based biomarkers in AD, and there are now several commercially available, adequately performing ultrasensitive plasma immunoassays detecting pTau181 that can discriminate between individuals with AD and controls with an AUC close to or over 0.9^5–7^. More recently, focus has switched to pTau217, i.e. tau phosphorylated at amino acid 217, which appears to discriminate better between individuals with AD pathology and controls due to a larger fold-change between the groups compared to pTau181^8^. pTau217 may furthermore become abnormal earlier than pTau181 in preclinical AD and have a stronger association with Aß and tau pathology as measured by PET^9,10^. The low concentrations of pTau217 in normal individuals and during preclinical AD make pTau217 a challenging biomarker to measure^8^ and there is still a lack of well validated pTau217 assays that are generally available. We evaluated a novel pTau217 S-PLEX assay developed by Meso Scale Discovery (MSD; Rockville, MD) employing ultrasensitive electrochemiluminescence (ECL) technology with additional signal enhancement^11^ in plasma from a sample (n = 131) of individuals with AD confirmed by CSF biomarkers and controls to determine assay precision and clinical accuracy.

## Materials and methods

### Study population

Plasma samples (n = 131) were selected from the Mass General Institute for Neurodegenerative Disease (MIND) biorepository, which includes plasma and CSF collected with research use consent at the time of diagnostic lumbar punctures at the Department of Neurology at Massachusetts General Hospital, and from the LifeSPAN biorepository, which contains plasma and CSF samples from healthy research participants. Clinical diagnoses were established by treating and research practitioners and confirmed in chart review by a senior neurologist (SEA) according to the 2011 National Institute of Aging-Alzheimer’s Association diagnostic criteria for AD^12^ and MCI due to AD^13^. Alzheimer's disease (AD) biomarker status was verified with CSF biomarkers measured by the MIND Biomarker Core (Aß42/40 ratio, pTau181, and tTau immunoassays; Euroimmun, Lübeck, Germany) available in 117 participants and/or Athena Diagnostics (ADmark Aß42, pTau181, and tTau; Marlboro, MA) analyzed as part of clinical care available in 80 participants. Participants with Euroimmun biomarkers were classified as AD if they had a low CSF Aß42/40 ratio (< 0.082) and increased pTau181 (> 41.8 pg/mL) and tTau (> 280 pg/mL) levels in CSF based on in-house derived thresholds defined in a cohort of 478 AD and control samples. Participants with Athena ADmark biomarkers were classified as AD if the Aß42/tTau Index (ATI) computed as ATI = Aß42/(240 + (1.18 ∗ (tTau))) exceeded 0.8^14^. The sample set included 61 samples from individuals with CSF (+) AD biomarkers and varying levels of cognitive impairment [34 dementia, 26 mild cognitive impairment (MCI), and 1 cognitively unimpaired]. 70 samples were from age and sex matched comparison individuals with CSF (−) AD biomarkers, consisting of 22 healthy volunteers (HC) and 48 individuals with other neurological diseases (OND). Further demographic and diagnostic details can be found in Table 1 and Supplementary Table 1.

**Table 1:** Clinical and Demographic Information.

		Diagnostic Groups	N	Age		Gender	CSF Aß42/40^b^	CSF ATI^c^	CSF pTau181^b^	CSF tTau^b^
				Mean (SD)	Range	Females N (%)	Mean (SD)	Mean (SD)	Mean (SD)	Mean (SD)
Batch I	AD	All combined	18	71.3 (8.2)	57–90	8 (44%)	0.54 (0.014)	0.51 (0.18)	103.7 (37.7)^c^	698 (271)^c^
		AD MCI	7	69.9 (7.5)	61–79	2 (29%)	0.060 (0.012)	0.64 (0.12)	89.0 (29.8)^c^	567 (159)^c^
		AD Dementia	11	72.2 (8.9)	57–90	6 (55%)	0.051 (0.016)	0.40 (0.08)	113.5 (40.8)^c^	784 (303)^c^
	CON	All combined^a^	18	70.6 (6.9)	57–82	7 (39%)	0.148 (0.039)	1.74 (0.56)	26.0 (7.3)	189 (65)
Batch II	AD	All combined	43	68.9 (10.0)	52–89	16 (37%)	0.052 (0.012)	0.44 (0.16)	114.2 (42.4)	518 (180)
		AD presympt	1	57 (NA)	NA	1 (100%)	0.064 (NA)	ND	125.6	544
		AD MCI	19	69.0 (7.9)	52–71	8 (42%)	0.050 (0.013)	0.43 (0.16)	104.8 (39.5)	501 (179)
		AD Dementia	23	69.4 (11.6)	54–89	7 (30%)	0.054 (0.012)	0.44 (0.16)	123.4 (45.6)	534 (191)
	CON	All combined	52	66.3 (8.7)	55–89	22 (42%)	0.127 (0.022)	1.56 (0.50)	25.6 (7.8)	196 (68)
		Healthy Controls	12	60.4 (4.5)	57–73	5 (42%)	0.133 (0.024)	ND	28.9 (10.4)	206 (77)
		OND^a^	40	68.0 (8.9)	55–89	17 (42%)	0.125 (0.020)	1.56 (0.50)	24.4 (6.4)	193 (65)

ATI, Aß42/tTau Index; AD, Alzheimer’s disease; CON, Controls; MCI, mild cognitive impairment; OND, Other neurological diseases.

^a^OND (Batch 1 and 2 combined): Normal pressure hydrocephalus (n = 20), immune mediated diseases (n = 6), neuropathies (n = 5), psychiatric disorders (n = 3), motor neuron diseases (n = 2), Lewy body dementia (n = 3), other (n = 6), not otherwise specified (n = 3). Specific diagnoses are listed in Supplementary Table 1.

^b^CSF Aß42/40 ratio, ATI, pTau181, and tTau measured using Euroimmun (n = 117) and/or Athena ADmark (n = 80) assays. 7 participants each in Batch 1 (6 AD/1 CON) and Batch 2 (4 AD/3 CON) had CSF ATN biomarkers measured only using Athena ADmark while the remaining participants had measurements done using Euroimmun assays alone or in combination with Athena ADmark.

^c^Measured using Athena ADmark assays.

The study was approved by the Institutional Review Board of Mass General Brigham (2015P000221 and 2018P001989) and all participants or their assigned surrogate decision makers provided written informed consent. All methods were performed in accordance with the ethical standards of the Declaration of Helsinki.

### Plasma sampling and analysis

Samples were collected in K_2_EDTA tubes, centrifuged and frozen within 2 h of collection, and stored at – 80 °C until use. Plasma pTau181 (catalog # K151AGMS) and pTau217 (catalog # K151APFS) levels were measured using ultrasensitive S-PLEX assay kits (MSD) employing a sandwich immunoassay format using monoclonal antibodies and electrochemiluminescence (ECL) detection in the MIND Biomarker Core. Calibrators for the pTau181 and pTau217 assays were prepared by using recombinant phosphorylated tau expressed in a mammalian system and confirmed by mass spectrometry to display phosphorylation at T181 or T217, respectively. Due to the lack of international standards, concentrations of calibrators were assigned via biochemical characterization and used to generate a calibration curve for sample quantitation. Lower limit of detection (LLOD) was defined as the concentration that provides a signal 2.5 standard deviations (SD) above the mean of the blank. Lower limit of quantification (LLOQ) was defined as the lowest calibrator concentration with a coefficient of variation (CV) < 20% and a recovery of 80–120%. Three quality control (QC) samples spanning the assay range and an 8-point calibration curve were included in duplicate on each plate. The samples were codified and randomized so that the technician performing the assay was blinded to any case information during testing and calculation of concentrations. Plasma samples were measured in duplicate using 25 uL of undiluted plasma per replicate.

CSF levels of Aß40, Aß42, pTau181, and tTau were measured using Euroimmun Beta-Amyloid (1–40) and (1–42), phospho-Tau/pTau(181) and Total-Tau ELISA assays (Lübeck, Germany) measured on a semiautomated Tecan Freedom Evo liquid handler (Männedorf, Switzerland) following manufacturer’s instructions.

### Statistical analysis

Statistical analysis was performed using STATA/SE version 16.1 (StataCorp, College Station, TX). Biomarker concentrations were natural log transformed to satisfy assumptions of normal distribution for statistical analysis while values in text and graphs are presented without log transformation. Values under the LLOQ were assigned the lowest quantifiable value of the assay. Differences between diagnostic groups were evaluated using two-sample t-test with equal variances. No adjustments for age or sex were performed as groups were well-balanced and no effects of age and sex were observed in initial analysis. Correlations between markers and with age were assessed with Pearson correlation coefficient. To determine classification utility of the biomarkers, area under the curve (AUC) values were computed using logistic regression models and confidence intervals calculated using bootstrapping. Comparisons between AUC curves were performed according to the DeLong algorithm. Optimal cut-points were estimated using the Youden index. A two-sided *p*-value < 0.05 was considered statistically significant.

## Results

### Assay performance

The assay performance of the pTau181 assay has been previously described^15^. The sensitivity of the pTau217 assay was determined from 6 plate-runs with 3 replicates each (18 measurements for each concentration level). Upper limit of quantification (ULOQ) was found to be 3,760 pg/mL with an inter-run CV of 7% and a total error (TE) of 14%. LLOQ was determined to be 1.84 pg/mL with an inter-run CV of 13% and a TE of 22%. LLOD was 0.395 pg/mL. The specificity of the pTau217 assay was tested against a non-phosphorylated Tau 441 and Tau with a mutation at site 217 from threonine to alanine (Tau 217A), preventing phosphorylation. Each of these was spiked into samples along with phosphorylated pTau217 calibrator and measured against control samples with no interferents. There was no interference with a median pTau217 recovery of 103% (range 91–115%). For calibrator recovery across all four plates, median calibrator signal recovery within the LOQ was 100% (range 87–114%).

Samples were measured in duplicate in two separate batches run two weeks apart using the same lot of reagents: Batch 1 contained 36 samples measured on one plate and Batch 2 contained 95 samples distributed over three plates, which were measured in parallel by the same technician. Concentrations of pTau217 were above LLOQ in all samples. Median intraplate CV for duplicates was 5.5% (range 0.3–15%) in Batch 1 and 4.6% (range 0.1–32%) in Batch 2. Three identical QC samples included on all three plates in Batch 2 resulted in a median interplate CV of 5.7% (range 0.3–13.4%) and normalized mean concentrations of the QC samples were 0.96–1.04 for the three plates. Eight batch-to-batch controls (five study samples and three QC samples) were included in both batches to determine interbatch variability. The median interbatch CV was 12.0% (range 1.0–17.9%). Concentrations of the batch-to-batch controls were slightly higher in Batch 1 (normalized median: 1.08, range 0.99–1.13) compared to Batch 2 (normalized median: 0.92, range 0.87–1.01), but concentrations were highly correlated (r = 0.99, *p* < 0.0001). pTau217 levels were normalized using the batch-to-batch controls using linear regression when results from the two batches were combined for further analysis.

### Patient characteristics

Samples were measured in two independent batches to identify and validate between-group differences in plasma levels of pTau217. The initial batch (Batch 1; n = 36) was performed to establish assay performance followed by a larger batch (Batch 2; n = 95) to determine between-group differences. Both batches contained age- and sex-matched individuals with AD and non-AD comparison cases consisting of HCs and individuals with a range of ONDs (Table 1; Supplementary Table 1). All individuals with AD had positive CSF ATN biomarkers to establish AD as the underlying pathophysiology for MCI and dementia. Preliminary analysis showed that pTau217 levels did not correlate with age among the controls (r = 0.18; *p* = 0.13) and there were no differences in pTau217 levels between females (mean ± SD: 5.0 ± 2.5 pg/mL) and males (5.9 ± 3.0 pg/mL; *p* = 0.19). Results did not change when the whole cohort was included in the analysis (data not shown). No adjustment for age and sex was thus performed in further analysis.

### Diagnostic performance of pTau217

**Figure 1:**
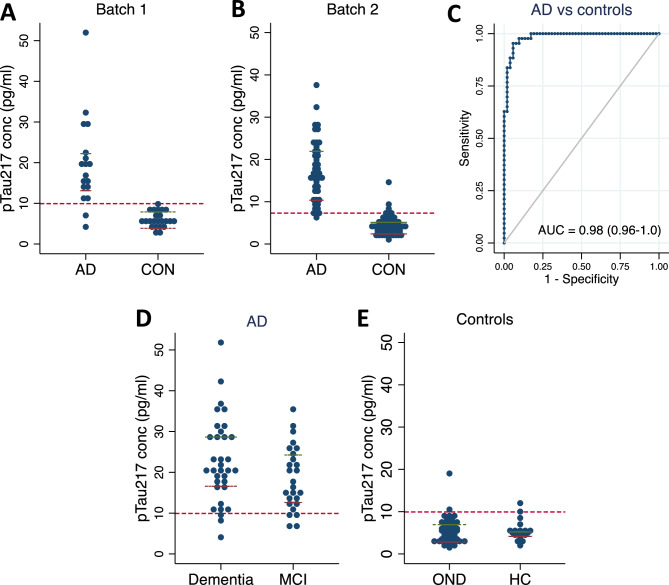
pTau217 plasma levels in individuals with Alzheimer’s disease (AD) and controls (CON). (**A**, **B**) Plasma pTau217 levels in individuals with AD and CON analyzed in two independent experiments: Batch 1 (**A**) and Batch 2 (**B**). (**C**) ROC curve for the classification of AD and controls using pTau217 in Batch 2. (**D**–**E**) Subgroup analysis comparing plasma pTau217 levels in individuals AD and different cognitive status [dementia or mild cognitive impairment (MCI); **D**] and controls with other neurological diseases (OND) or healthy volunteers (HC; **E**). Red dashed lines represent the optimal cut points between AD and controls as defined by the Youden index.

Results from the initial batch demonstrated that pTau217 concentrations were 3.3-fold higher in AD (19.7 ± 11.0 pg/mL) compared to controls (6.0 ± 2.0 pg/mL; *p* < 0.0001; Fig. 1A). The two diagnostic groups were well separated with an AUC of 0.94 (95% CI 0.85–1.03) in the initial batch. This excellent performance was repeated in the larger set of samples in the second batch where pTau217 levels were 3.9-fold higher in AD (17.3 ± 7.4 pg/mL) compared to controls (4.4 ± 2.4 pg/mL; *p* < 0.0001; Fig. 1B). The AUC to discriminate between CSF biomarker-defined AD and comparison samples was 0.98 (95% CI 0.96–1.0) in the second batch (Fig. 1C). The optimal cut-point between the two groups as defined by the Youden index was 7.3 pg/mL (J = 0.90), corresponding to 95% sensitivity and 94% specificity at the cut-point. Subgroup analysis did not show any differences in pTau217 levels between individuals with AD and different cognitive status (dementia: 22.9 ± 10.3 pg/mL; MCI: 18.6 ± 7.7 pg/mL; *p* = 0.14; Fig. 1D) or between OND (5.5 ± 3.0 pg/mL) and HC (5.4 ± 2.4 pg/mL; *p* = 0.9; Fig. 1E).

### pTau217 outperforms pTau181 in diagnostic classification

**Figure 2:**
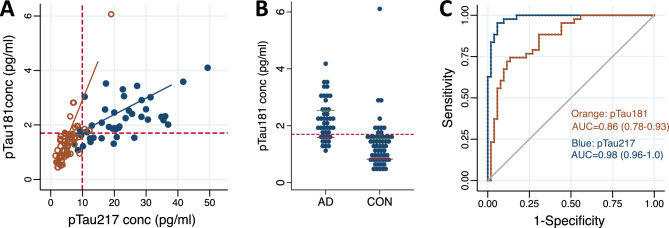
Comparison of plasma pTau181 and pTau217 assays. (**A**) Correlation between plasma levels of pTau181 and pTau217 in individuals with AD (blue filled circles) and controls (orange open circles). (**B**) Plasma pTau181 levels in individuals with AD and controls (CON). (**C**) ROC curve for the classification of AD and controls using pTau181 (orange) and pTau217 (blue). Red dashed lines represent the optimal cut points between AD and controls as defined by the Youden index.

pTau181 was measured in the second batch to compare the diagnostic accuracy between the two analytes. This showed that pTau181 and pTau217 levels were highly correlated both in the whole cohort (r = 0.80, *p* < 0.00001) and when limiting the analysis to individuals with AD only (r = 0.63; *p* < 0.0001; Fig. 2A). While individuals with AD had higher pTau181 concentrations compared to controls (2.2 ± 0.7 vs. 1.3 ± 0.8 pg/mL, *p* < 0.0001; Fig. 2B), this only represented a 1.7-fold difference compared to the 3.9-fold increase observed for pTau217. The AUC to discriminate between AD and controls for pTau181 was 0.86 (95% CI 0.78–0.93), which was significantly lower than using pTau217 (*p* = 0.0002; Fig. 2C). The optimal cut-point between AD and controls for pTau181 was 1.7 pg/mL (J = 0.59) corresponding to 74% sensitivity and 84% specificity at the cut-point.

### Correlations with CSF AD biomarkers

**Figure 3:**
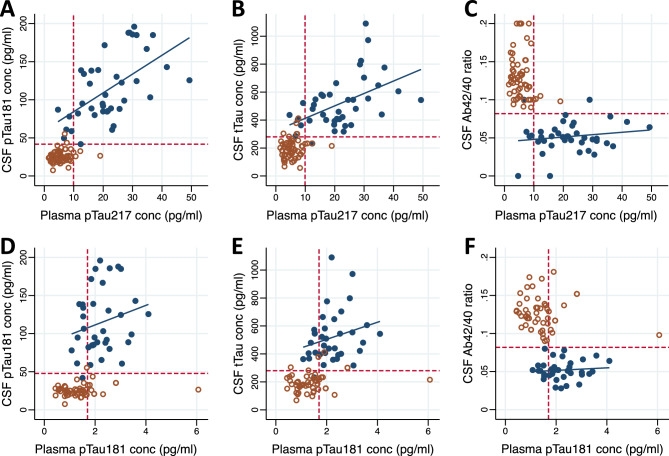
Correlations between plasma and CSF biomarkers. Correlations between plasma pTau217 (top) or pTau181 (bottom) and CSF pTau181 (**A**, **D**), tTau (**B**, **E**), and Aß42/40 ratio (**C**, **F**). Blue filled circles = Alzheimer’s disease (AD); Orange open circles = Controls. Blue lines show Pearson correlation between the plasma and CSF markers among individuals with AD. Red dashed lines represent the optimal cut points between AD and controls as defined by the Youden index.

Plasma levels of pTau217 correlated with CSF levels of pTau181 and tTau as measured using Euroimmun assays both in the whole cohort (CSF pTau181: r = 0.82; *p* < 0.0001; CSF tTau: r = 0.78; *p* < 0.0001) and within the individuals with AD (CSF pTau181: r = 0.60; *p* < 0.0001; CSF tTau: r = 0.48; *p* < 0.002; Fig. 3A,B). Plasma levels of pTau217 also correlated with the CSF Aß42/40 ratio in the whole cohort (r = − 0.63; *p* < 0001), but not within the individuals with AD (r = − 0.055; *p* = 0.74; Fig. 3C). Plasma levels of pTau181 correlated with CSF levels of pTau181 (r = 0.57; *p* < 0.0001), tTau (0.54; *p* < 0.0001), and the Aß42/40 ratio (r = − 0.51; *p* < 0.0001) within the whole cohort, but these correlations were lost when limiting the analysis to individuals to AD only (CSF pTau181: r = 0.26; *p* = 0.13; CSF tTau: r = 0.29; *p* = 0.09; CSF Aß42/40 ratio: r = 0.04; *p* = 0.83; Fig. 3D–F).

## Discussion

There is an urgent need for widely available immunoassays for plasma pTau217 to evaluate AD pathology in individuals with cognitive symptoms. While a growing number of publications suggest that plasma pTau217 performs similarly to well-established CSF biomarkers and PET imaging to determine amyloid pathology and provide diagnostic and prognostic information^16^, a significant limiting factor thus far has been that both the two most extensively studied assays, the Simoa immunoassay developed by Janssen Research and Development^17^ and the immunoassay developed by Lilly Research Laboratories^10^ using the MSD® ECL platform, are homebrewed assays developed in labs or clinical research organizations (CROs) using proprietary antibodies and have not been commercially available. More recently, the ALZpath pTau217 assay^18^, another Simoa immunoassay developed by ALZpath Inc., has been released commercially but there is still a need for additional well validated commercial assays. These assays need to be affordable and available on platforms that are easily implemented not only in large, centralized settings, but also in community settings and smaller laboratories with more limited resources.

Here, we evaluated the technical and diagnostic performance of a novel ECL-based ultrasensitive plasma pTau217 S-PLEX assay developed by MSD and demonstrated that the assay performance was excellent with an AUC of 0.98 to differentiate between individuals with AD confirmed by CSF biomarkers and controls and 3.9-fold differences in pTau217 levels between the two groups. While difficult to compare between study cohorts, these performance characteristics are comparable or superior to the ability of previously published plasma pTau217 assays to discriminate between patients with and without positive CSF AD biomarkers^7^. A round robin study is currently ongoing to determine the performance of the currently available pTau217 assays in identical sample cohorts in a direct head-to-head comparison. The new MSD® pTau217 S-PLEX assay performed better than plasma pTau181 in discriminating between AD and controls primarily due to the larger fold-differences for the pTau217 assay resulting in less overlap between the groups as has previously been demonstrated for other pTau217 assays^5,7,19,20^. There was a good correlation between plasma pTau217 and CSF pTau181 and tTau both in the whole cohort and the participants with AD. In contrast, we did not see any correlation between plasma pTau217 and the CSF Aß42/40 ratio in participants with AD even though there was an excellent qualitative ability to differentiate CSF Aß42/40 positive and negative individuals reflecting that the trajectory for CSF Aß42/40 accumulation tend to plateau once Aß positivity as determined by PET has been reached^21^.

While more research still is needed to fully understand how pTau217 is affected by comorbidities and factors such as race and socioeconomical background before plasma pTau217 measurements can be implemented in primary care settings to diagnose AD, the rapid development of ultrasensitive assays to measure pTau217 in blood promises to transform clinical practice and clinical research. Plasma pTau217 measurements can be envisioned to be used in screening of healthy individuals at risk for AD, to aid in the differential diagnosis of individuals with cognitive decline, and to monitor treatment responses of disease modifying treatments^22^. The recently approved anti-Aß therapies furthermore stresses the need to be able to determine if an individual has AD pathology or not in a timely manner. The current appropriate use recommendations for blood biomarkers in AD propose a three-tiered approach where properly validated plasma pTau assays can be used to screen individuals into low, medium, and high probabilities for AD where confirmatory testing by CSF biomarkers or PET imaging primarily will only be required in the medium probability group limiting the need for more invasive and expensive testing^3^.

In conclusion, our results show that the novel MSD pTau217 S-PLEX assay performs well and in par with other pTau217 assays. A larger study on a cohort with clinically well-characterized samples and longitudinal follow-up is currently ongoing to confirm the assay’s clinical performance. Well validated plasma pTau217 assays have shown promise for use as simple, accessible, and scalable tests for AD pathology in the screening of individuals with cognitive decline.

### Supplementary Information

Supplementary Table 1.

## Data Availability

Anonymized data not published within this article will be made available on reasonable request from any qualified investigator by the corresponding author (PK).
