# Supplementary material for: Clinical evaluation of a novel plasma pTau217 electrochemiluminescence immunoassay in Alzheimer’s disease
Source: Sci Rep. 2024 Jan 5;14:629. doi: 10.1038/s41598-024-51334-x (PMC10770381; doi:10.1038/s41598-024-51334-x)
Supplement: Supplementary file 1 — Supplementary Table 1. [file 41598_2024_51334_MOESM1_ESM.pdf]

Supplementary Table 1

Diagnostic information on study participants with other neurological diseases (OND)

Category	Diagnosis	Batch	n
Normal Pressure Hydrocephalus	Normal pressure hydrocephalus	1	5
	Normal Pressure Hydrocephalus	2	15
Immune Mediated Diseases	Autoimmune encephalopathy	2	1
	Cerebellar degeneration, autoimmune	2	1
	Meningitis, resolved	2	1
	Multiple sclerosis	2	1
	Polyneuropathy, demyelinating	2	1
	Uveitis	2	1
Neuropathies	Peripheral neuropathy	1	1
	Peripheral neuropathy, small fiber	1	1
	Polyneuropathy	1	1
	Diabetic autonomic neuropathy	2	1
	Trigeminal neuralgia	2	1
Psychiatric Disorders	Bipolar disorder	2	2
	Functional Neurological Disorder	2	1
Lewy Body Dementia	Parkinsons Disease	2	2
	Dementia with Lewy bodies	2	1
Motor Neuron Diseases	Primary lateral sclerosis	2	2
Other	Atypical parkinsonism	2	1
	Cerebrovascular disease	2	1
	Migraine headaches	2	1
	Spinocerebellar ataxia type 6	2	1
	Temporal lobe epilepsy	2	1
	Vestibular gait disorder	2	1
NOS	MCI NOS	2	3
